# A Point-of-Care Device for Molecular Diagnosis Based on CMOS SPAD Detectors with Integrated Microfluidics

**DOI:** 10.3390/s19030445

**Published:** 2019-01-22

**Authors:** Joan Canals, Nil Franch, Oscar Alonso, Anna Vilà, Angel Diéguez

**Affiliations:** Electronic and Biomedical Engineering Department, University of Barcelona, 08028 Barcelona, Spain; nfranch@el.ub.edu (N.F.); oalonso@el.ub.edu (O.A.); avila@el.ub.edu (A.V.); adieguez@el.ub.edu (A.D.)

**Keywords:** fluorescence lifetime, low-cost, molecular diagnosis, microfluidics, point-of-care, CMOS, single-photon avalanche diode, laser diode

## Abstract

We describe the integration of techniques and technologies to develop a Point-of-Care for molecular diagnosis PoC-MD, based on a fluorescence lifetime measurement. Our PoC-MD is a low-cost, simple, fast, and easy-to-use general-purpose platform, aimed at carrying out fast diagnostics test through label detection of a variety of biomarkers. It is based on a 1-D array of 10 ultra-sensitive Single-Photon Avalanche Diode (SPAD) detectors made in a 0.18 μm High-Voltage Complementary Metal Oxide Semiconductor (HV-CMOS) technology. A custom microfluidic polydimethylsiloxane cartridge to insert the sample is straightforwardly positioned on top of the SPAD array without any alignment procedure with the SPAD array. Moreover, the proximity between the sample and the gate-operated SPAD sensor makes unnecessary any lens or optical filters to detect the fluorescence for long lifetime fluorescent dyes, such as quantum dots. Additionally, the use of a low-cost laser diode as pulsed excitation source and a Field-Programmable Gate Array (FPGA) to implement the control and processing electronics, makes the device flexible and easy to adapt to the target label molecule by only changing the laser diode. Using this device, reliable and sensitive real-time proof-of-concept fluorescence lifetime measurement of quantum dot Qdot^TM^ 605 streptavidin conjugate is demonstrated.

## 1. Introduction

Nowadays, fluorescence-based techniques have become among the most widely used methods in clinical analysis and biomedical diagnosis [1] and have been applied in the field of high-throughput biotechnology for Fluorescence Activated Cell Sorting (FACS) and High-Throughput Screening (HTS) thanks to their accuracy, sensitivity (single molecule detection), and targeted labelling of biological samples [2].

In particular, fluorescence measurements can provide information not only on the specific molecular makeup of a sample, but also on the local environment surrounding the fluorescence molecule or fluorophore (such as pH, ion concentrations, etc.), which give value to the fluorescence based techniques a powerful analysis tool [3,4,5,6]. However, the fluorescence techniques based on intensity measurements are prone to misinterpretation due to their dependence on parameters such as excitation light intensity and fluorophore concentration. In contrast, time-resolved fluorescence techniques can address the limitations of intensity-based measurements by time resolving the fluorescence decay or lifetime, which is an intrinsic molecular property, thus independent of the fluorophore concentration and the excitation intensity [7]. Moreover, the fluorescence lifetime improves the specificity of the fluorescence measurement by time domain discrimination. This allows for the differentiation of fluorophores with overlapping emission spectra but different lifetimes [8], as well as to discern the light of interest from the background light due to autofluorescence of the biological sample, which can distort quantitative intensity based measurements [9].

Thus, many efforts have been done in recent years to develop time-resolved methods as Time Resolved Fluorescence Spectroscopy (TRFS) and Fluorescence Lifetime IMaging (FLIM), which allow in vivo characterization and diagnosis of biological samples [10,11,12,13,14,15]. However, conventional equipment needed to perform fluorescence lifetime measurements—a pulsed laser; a complex opto-mechanical system with lens and filters, generally a microscope; and a photon transducer such as a CCD camera or photomultiplier tube [16,17]—is bulky and expensive, and this relegates it to research laboratories.

On the contrary, Point–of-Care (PoC) applications, are aimed at performing analytical or diagnostic tests near the site of interest, such as a patient in a hospital or even on the field, in order to provide on-site results to the operator [18]. In the last decade, noticeable progress has been made towards the miniaturization of fluorescence-based instruments, in an effort to reduce both cost and size. Considerable gains have been achieved thanks to the integration of microfluidics [19,20,21,22] and sensing microtechnologies like Complementary Metal-Oxide-Semiconductor Single-Photon Avalanche Diodes (CMOS SPADs) [23,24,25,26], but the obtained prototypes either require bulky optical setups with filters, lens, or optical fibers to guide the light [2,16,17,25,27,28,29,30] or suffer loss of sensitivity [26] or increased measurement time [8]. Very compact analysis systems have been reported [31,32,33], but they still require a filter to subtract the excitation light source and an expensive Time Correlated Single Photon Counting (TCSPC) card to build the histogram of the decay time.

**Figure 1:**
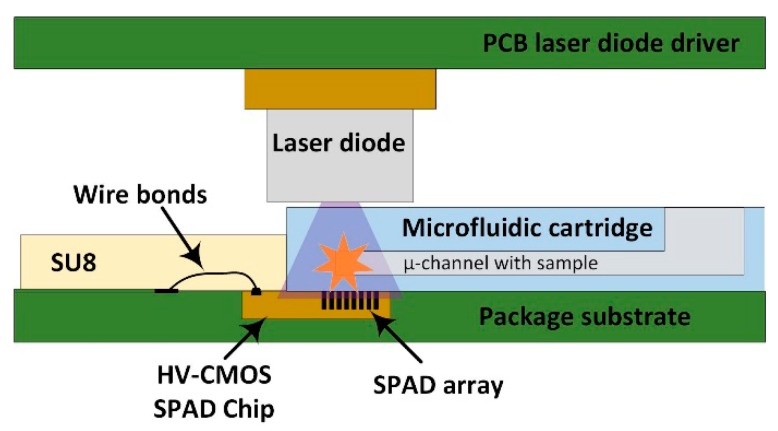
Cross-sectional sketch of the stack up of the system. The sample is introduced in one of the microchannels of the microfluidic cartridge, which is directly positioned on top of the SPAD array, where the wire bonds encapsulation with SU-8 prevents it from entering further.

In this work, we describe a compact general-purpose portable device, aiming to overcome the limitations of current PoC molecular diagnosis systems. In order to obtain a sensitivity comparable to actual optical instruments while a diagnosis time of a few seconds, different microtechnologies have been combined. In particular, the system integrates an array of ultra-sensitive HV-CMOS SPAD detectors with a custom interchangeable polydimethylsiloxane (PDMS) microfluidic cartridge to insert the sample, and a low-cost pulsed UV laser diode as excitation light source (Figure 1). The proximity between the sample in the microfluidic cartridge and the SPAD sensor together with the gated operation mode of the sensor [34] makes unnecessary the use of lenses and optical filters for long lifetime fluorophores, such as quantum dots (QD). Moreover, the SPAD array sensor chip is packaged using a custom developed SU-8 encapsulation able to protect the wire bonds of the chip, while at the same time acting as physical barrier for the microfluidic cartridge. The combination of the sensor packaging with the custom microfluidic chip allows placing the sample at less than 200 μm from the sensor chip surface without requiring any alignment procedure. The control and processing electronics are synthetized on a FPGA, where the TCSPC technique [35] is implemented to measure the decay profile of the fluorescence with sub-nanosecond resolution. The time histogram build within the FPGA can then be read via a USB interface or directly plotted on a display. The use of a FPGA and a laser diode makes the system configurable in such a way that it can be easily adapted to the target label molecule and measurement needs.

The following sections introduces the measuring technique adopted, followed by a description of the device, and finally, a complete characterization of the device, as well as fluorescence lifetime measurements of several concentrations of quantum dot, Qdot^TM^ 605 Streptavidin Conjugate (QD605) from Thermo Fisher Scientific (Waltham, MA, USA), are reported.

## 2. Materials and Methods

### 2.1. Materials

In Table 1 the commercially available elements used to construct and test the PoC-MD are listed. The SPAD array chip is included. It was custom designed and fabricated with a multi-project wafer in a 0.18 HV-CMOS process. The basic electronic components are not listed for simplicity. The initial investment is high due to the raw materials such as SU8, PDMS, CW2400, and the FPGA development board. Nevertheless, the final cost of the prototype is approximately 960 €, which is much more affordable than commercial bulky equipment.

PDMS was selected to manufacture the microfluidics cartridge because it has good optical properties in the range of NUV-VIS-NIR (400–1000 nm) and presents a low autofluorescence emission, which is invariant to illumination [36]. As a substrate, a glass cover slip of 150 μm (12460S from Thermo Fisher Scientific, Waltham, MA, USA) is selected to guarantee good transmission of the light emitted by the QD to the detector.

### 2.2. Measuring Technique

**Figure 2:**
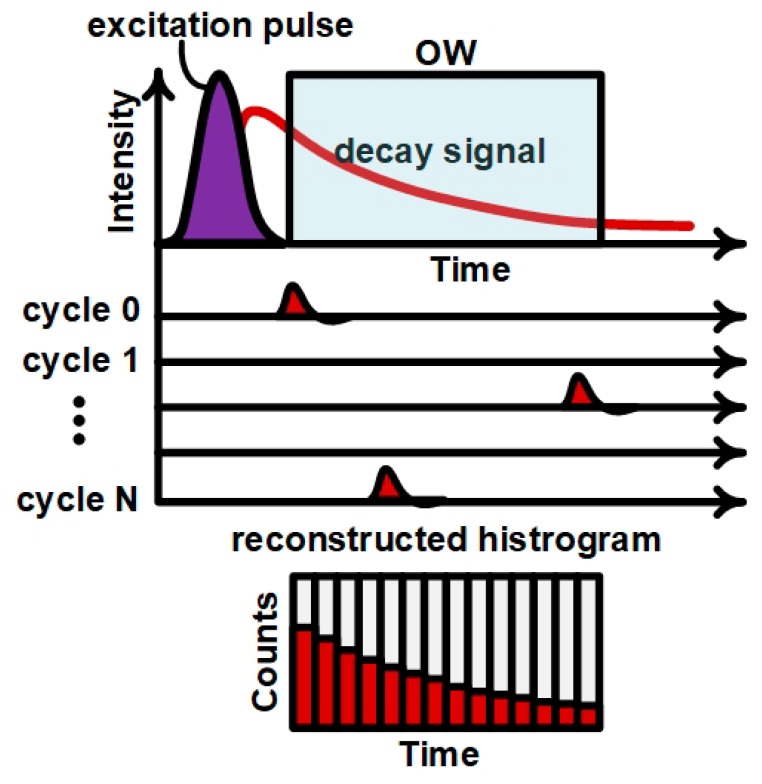
Measurement procedure applying the time gating to cut off the laser.

TCSPC is illustrated in Figure 2. The sample is repetitively excited by the pulsed light source. After each excitation pulse, fluorescence emission occurs and only a single photon of the many emitted can be detected by the SPAD, which remains inhibited after the detection. The arrival time of this photons is measured and follows the probability distribution of the fluorophore emission, which is then reconstructed in the histogram generated over multiple excitation/emission cycles. On the other hand, the SPAD sensor is only activated during a programmable time (the observation window, OW) that by a user-defined time with a resolution of 68 ps. The time offset between the laser pulse and the OW allows to suppress the excitation light as well as unwanted background signals (like auto-fluorescence from cells or media) from the OW without using the optical filters typical of fluorescence measurement setups [37].

The measurement begins by setting the OW, which is delayed with respect to the laser trigger, thus preventing the excitation light from saturating the SPAD sensor. If an avalanche occurs during the OW, the time at which this event occurred is captured by the Time-to-Digital Converter (TDC) of the system controller and it is stored in the histogram memory. The TDC has a timing resolution of 68 ps with a measurement range up to 420 ns, and the time values are quantized in bins, corresponding to respective memory positions. In order to have a significant statistical population of measurements, the measurement repeated a configurable number of times. Next, a histogram of events per time bin is built to show the decay profile. For a single fluorophore, as a first approximation, the histogram appears as an exponential decay with lifetime *τ* [38],

(1) $$I(t)=I_{0}e^{-t/\tau }.$$

The PoC-MD allows the generation of a Dark Count Rate (DCR) profile of the sensor array in a dark environment by masking the laser trigger, useful to select the SPAD-microchannel pair with the best Signal-to-Noise Ratio (SNR). The noise measurement is done like a lifetime measurement but without pulsing the laser diode. Once the histogram of the dark count noise is generated, the DCR is calculated as follows:

(2) $$\mathrm{DCR}(\mathrm{Hz})=\frac{\mathrm{Number}\;\mathrm{of}\;\mathrm{counts}}{\mathrm{Number}\;\mathrm{of}\;\mathrm{Measurements}\times \mathrm{Time}\;\mathrm{of}\;\mathrm{OW}}$$

In the same way, the system can perform measurements of fluorescence intensity by adding the counts of the observation window while pulsing the laser.

### 2.3. Device Implementation

#### 2.3.1. SPAD Sensor Chip Design

The sensor geometry (SPAD array size, array configuration, array-contact pad layout, and SPAD size) was designed together with its packaging and the microfluidics cartridge as a whole, in order to allow the straightforward stacking of the microfluidic cartridge and the die without any alignment procedure. As seen in Reference [32], the light resulting from the fluorescence in one microchannel arrives to more than one pixel, making unnecessary the use of a 2-D array. Consequently, a 1-D array gave enough information, which simplifies the readout and control electronics and reduces the number of connections and the power consumption.

**Figure 3:**
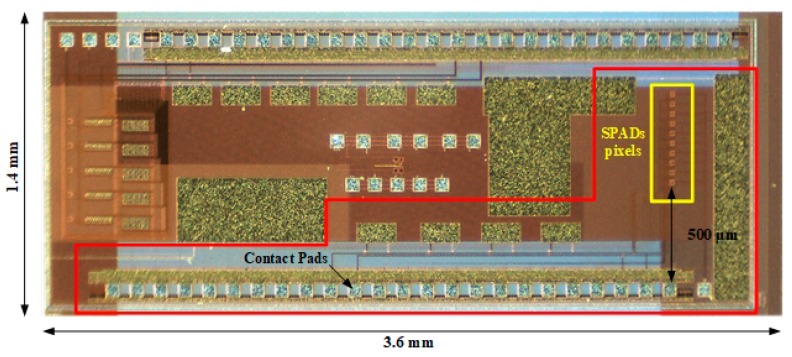
Photography of the ASIC with the implemented circuit highlighted in red and the 10 SPADs pixels highlighted in yellow. The first SPAD pixel is situated at 500 μm from the edge of the contact pads.

The SPAD sensor array chip is implemented in a standard 0.18 μm HV-CMOS process. The 3.6 mm × 1.4 mm chip (Figure 3), contains a 1-D array of 10 SPAD detectors with 50 μm pitch (highlighted in red in the figure), along with other test structures. The active area of each detector is 8 μm in diameter. Each SPAD pixel has its digital output but the control signals and the bias voltage are common to all the SPADs. All the connections of the circuit are placed on the long side of the chip and the 1-D SPAD array is placed perpendicular to them, with the first SPAD at 500 μm from the edge of the bonding pads. This configuration allows a simple microfluidic design with good tolerances for its manufacture, as well as for the packaging of the sensor itself.

The active area of the pixels is kept low to have a low dark count noise, which is proportional to the active area and the temperature [39]. The only reason to use SPADs with larger active area would be to easily align them, but this is solved by the use of 1-D array and the sensor packaging, which ensures that at least one SPAD is covered by a microfluidic channel of 100 μm width.

**Figure 4:**
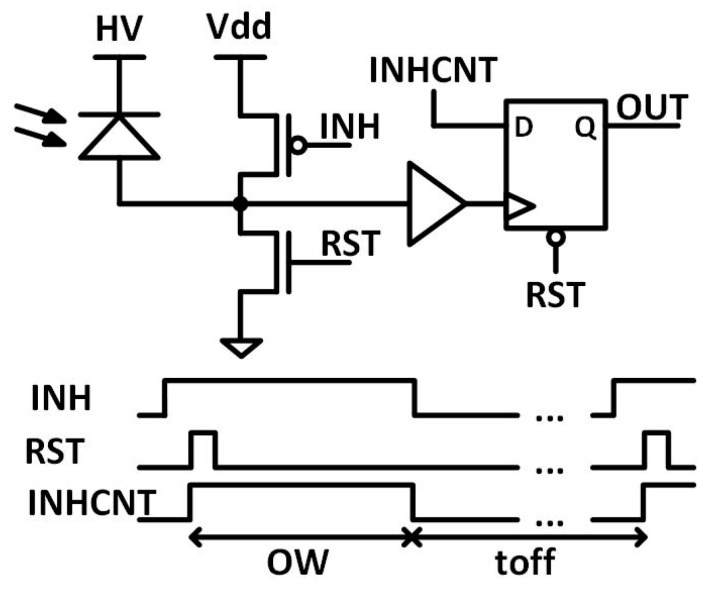
SPAD pixel schematic and time diagram of SPAD control signals.

The SPADs design is based on the work by Reference [40], but with a different readout electronics (Figure 4). The n+ cathodes are biased at a positive high voltage (V_HV_) beyond its breakdown voltage (V_BD_, approximately 11.7 V) by an overvoltage (V_OV_) to operate in Geiger mode, V_HV_ = V_BD_ + V_OV_. The avalanches are sensed at the p+ anode due to its lower intrinsic capacitance to ground, which is beneficial in reducing the timing response, as well as the afterpulsing probability [41]. The SPADs are operated in gated mode to partially eliminate the after-pulsing and reduce the probability to detect dark counts instead of desired events [42]. Gated operation is accomplished by three external signals RST, INH and INHCNT: INHCNT inhibits the readout electronics, INH inhibits the SPAD sensor by setting the bias voltage below its breakdown voltage, and finally the RST discharges the sensing node and prepares it to measure again. Figure 4 shows the time diagram of the SPADs control signals. The pulse width of INHCNT signal determines the observation window of the SPAD. The duration of INH at low level determines the off time, which should be held relatively large to avoid the afterpulsing. Nevertheless, these sensors show almost negligible after-pulsing (<0.2%), which is in line with other works [43] and can be avoided almost completely with a 200 ns of off time. The timing jitter for these sensors is expected to be below 80 ps for 1.4 V of overvoltage and 8 μm SPAD size, accordingly with similar SPADs [43].

#### 2.3.2. Sensor Packaging

The SPAD array sensor chip is mounted on a specific Printed Circuit Board (PCB) that acts as a package substrate thanks to a recess into which the chip can be inserted. The recess is 50 μm deeper than the chip height (approximately 350 μm) so that when the chip is glued with a conductive epoxy (CW2400 from Chemtronics Circuit Works) into the recess, its surface is flush with the PCB, creating a planar surface for the microfluidics. The combined PCB and chip are subjected to a dehydration bake at 150 °C for 15 min and wire bonded, keeping the wire bonds as flat as possible, in order to ensure a good coating.

**Figure 5:**
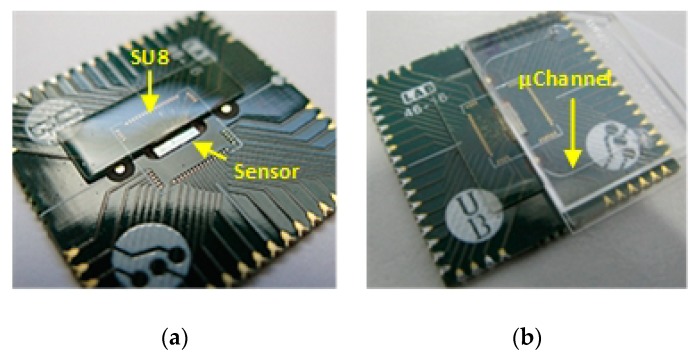
(**a**) Fully encapsulated SPAD Sensor Array chip using SU8 to protect the wire bonds; (**b**) SU8 wire bonds protection as stop for the microfluidic chip.

SU-8 and photolithography are then used to encapsulate the wire bonds, and created bulge will be used as a physical limit to place the microfluidic chip (see Figure 5). SU-8 is spin coated onto the board at 1000 rpm for 30 s, using a process based on the recommended conditions in the SU-8 datasheet [44]. The soft-bake is performed using temperature ramping from room temperature to 65 °C and holding for 30 min, increasing to 95 °C and maintaining for 90 min and slowly cooling down to 40 °C before exposure. The same scheme is followed for the post-exposures bake but changing the times to 10 and 30 min, respectively. These heating by temperature ramping and slowly cooling down, reduce the stress and any adhesion problems caused by the different thermal expansion rates of the SU8, PCB, and chip [45]. The final SU8 cap (Figure 5a) covers a surface of 4.6 mm × 12 mm and extends 200 μm over the chip from the edge of the pads, leaving a margin of 300 μm to locate microfluidic cartridge. The resultant encapsulation is a 450 μm tick, which is enough to act as for the microfluidic chip (Figure 5b).

#### 2.3.3. Microfluidic Cartridge Fabrication

The microfluidic cartridge was fabricated in a standard PDMS soft-lithography process [46,47] using a SU-8 mold fabricated by photolithography [48,49]. The cartridge structure was obtained by mixing the base and curing agent together at a 10:1 ratio (w/w), degassing, pouring into a Petri dish with the microchannel mold therein, degassing again and curing. The resultant PDMS was cut and sealed irreversibly with a glass coverslip (12460S from Thermo Fisher Scientific) of 150 μm thickness and 24 mm × 60 mm shape, by exposing both sides to be bonded to an air plasma treatment at 30 W for 60 s.

**Figure 6:**
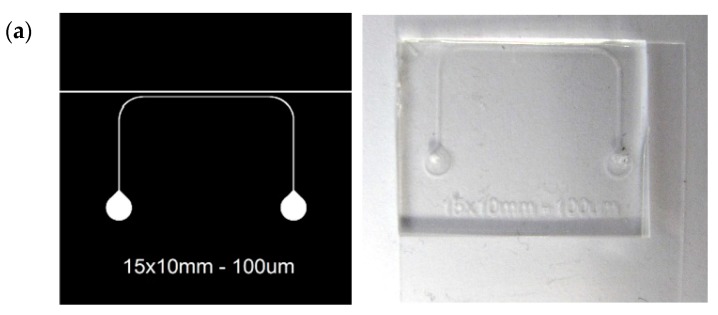

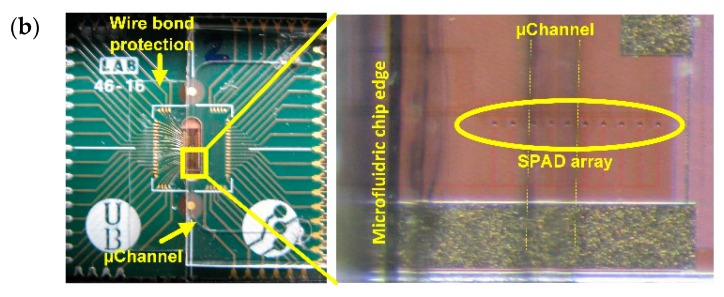
(**a**) Microfluidic design and final cartridge; (**b**) Microfluidic cartridge over the encapsulated chip with a detail of the microchannel over the SPAD array.

The structure and geometry of the microfluidic cartridge was fixed by the geometry of the SPAD sensor chip, its packaging and the selected substrate to seal the microfluidics. Figure 6a shows the design of the microfluidic chip, which consists of a single channel 100 μm wide and 100 μm high together with a cutting guide (150 μm wide) sited 300 μm away from the microchannel to ensure its position over the SPAD sensor array within the tolerance margins (Figure 6b). Being spaced by 42 μm, this microchannel width guarantees that at least one sensor is fully covered by the channel (Figure 6c).

A thickness of 2 mm of PDMS is sufficient to firmly connect the inlet and outlet tubes while allowing the excitation light source close enough to the sample, to prevent the need of focusing optics. The thickness of the PDMS slice is controlled by the volume of PDMS poured on the mold using a syringe, which produces a variation of ±200 μm. The coverslip substrate allows placing the sample close enough to the SPADs (~200 μm) to have a good detection and provides good cartridge handling.

#### 2.3.4. Excitation Source

In order to obviate the use of optical filters, a light source fast enough to be switched off, before the detector is gated on is unavoidable. While it has been reported that low-cost LEDs can be pulsed to measure the fluorescence decay times [15,26,50], a more powerful light source might increase the intensity of the fluorescence signal at low concentrations and compensate the signal loss during the elapsed time between the excitation light pulse and the beginning of the observation window.

**Figure 7:**
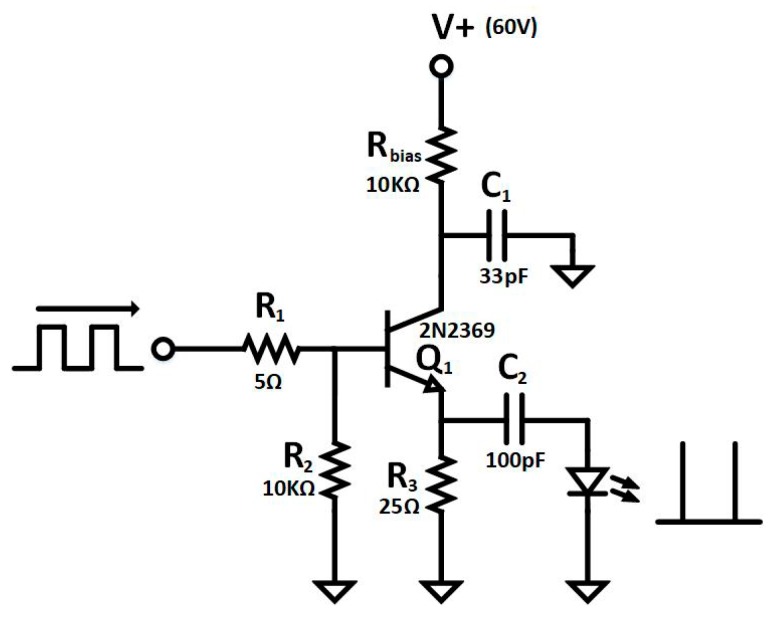
Simplified schematic of avalanche pulse generator.

In our application, the sample excitation is achieved using a L405P150 laser diode from Thorlabs, a 405 nm diode with an output power of 150 mW. The excitation wavelength was chosen to be 405 nm since this provides a close match with the excitation wavelength of many commonly used fluorophores including the QD605. Figure 7 shows the driving circuit used to generate sharp short pulses in order to measure short lifetimes. This circuit is based on the avalanche breakdown pulse operation of the 2N2369 small signal NPN transistor [51,52,53,54], which exhibits avalanching with performance similar to transistors dedicated to avalanche at a lower price [15]. The amplitude and width of the generated electrical pulses can be tuned by V+ and C1 capacitor. The implemented circuit emits short electrical pulses of 1.6 ns FWHM with 10 V amplitude into a load of 50 Ω with a repetition rate of 10 kHz, which is enough to drive the laser diode and generate sharp light pulses to stimulate the emission of the sample.

#### 2.3.5. System Controller

The system controller is based on a previous work [55], now combined with the SPAD array described in this paper, and implemented using the Zynq7020 All Programmable System-On-Chip (AP SoC) from Xilinx. While the controller can be integrated in the same chip as the SPADs [32,56,57,58,59], usually an external processor or FPGA is still required, whether for post processing the data, configuring the device, or for showing the results via PC or display. The use of Zynq 7020 AP SoC is preferable even with the increased cost that it supposes, because it introduces great flexibility brought by connecting directly a software platform with an FPGA. This solution enables the construction of an independent system with a high flexibility by reprogramming the hardware or software.

The AP SoC consists of a processing system (PS) based on a dual-core Cortex-A9 ARM processor, and a programmable logic (PL) Artix-7 FPGA. The PS is in charge of post processing data and managing the communication with a PC, by receiving the experiment configuration parameters and sending the resulting histogram. The PL implements the measurement technique described in Section 2.2.

#### 2.3.6. System Configuration

**Figure 8:**
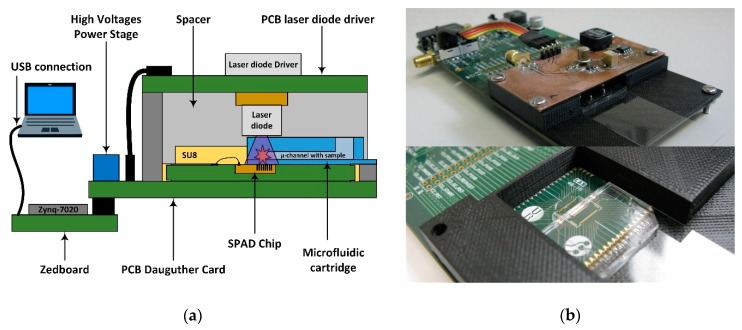
(**a**) Scheme diagram of the complete PoC-MD system with the detailed system stack; and (**b**) complete PCB daughter card with a detail of microfluidic cartridge guide over the SPADs sensor.

A dedicated PCB daughter card has been designed to hold the system stack, which consists of a sandwich structure including the SPAD sensor chip, a microfluidic cartridge and an UV laser diode (Figure 8). The packaged SPAD sensor chip is situated on top of the daughter card. The laser diode and its driver are implemented on an auxiliary PCB, connected to the daughter card, and placed over the SPAD sensor chip facing the laser diode against the sensor array. The minimum distance between the laser diode and the SPADs detectors is determined by the microfluidic cartridge height (2 mm). A plastic spacer is designed to house the stacked system, setting the distance between the laser diode and the SPAD array at 3 mm. An opening on one side of the spacer the custom microfluidic cartridge to be inserted and guided over the SPAD array until it reaches the SU8 stop of the sensor packaging which ensures its correct position.

The Zynq7020 AP SoC system controller is mounted on a Zedboard development board connected to the daughter card via FMC connector. This electrical connection includes all the digital signals as well as the power and ground supplies, except the high-voltages bias required by the SPADs sensors and laser diver, which are generated from the 3.3 V supply of the Zedboard by a power stage implemented on the daughter card. The Zedboard receives the measurement configuration parameters, and at the end of the measurement sends the generated histogram to a PC via USB for visualization and lifetime extraction. Figure 8 shows the complete PoC-MD configuration and the designed daughter card with the system stack-up.

## 3. Results

### 3.1. Sensor Characterization

The device performance is determined by the main parameters characterizing individual SPADs, i.e., noise through Dark Count Rate (DCR) and sensitivity through Photon Detection Probability (PDP) [60]. DCR is measured as the rate of random pulses due to thermally generated carriers. It has a high variance due to its dependence on the number of traps in the diode.

**Figure 9:**
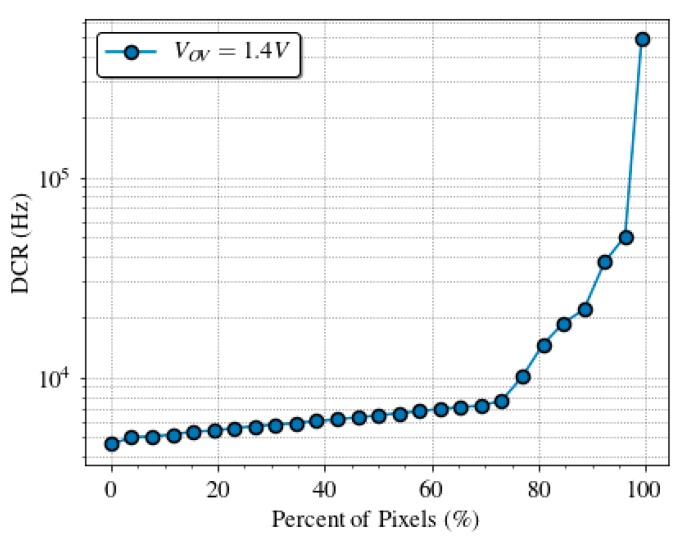
Dark count rate distribution measured across the SPAD array of 13 chips at 1.4 V of overvoltage and room temperature with a maximum relative error of 0.5%.

**Figure 10:**
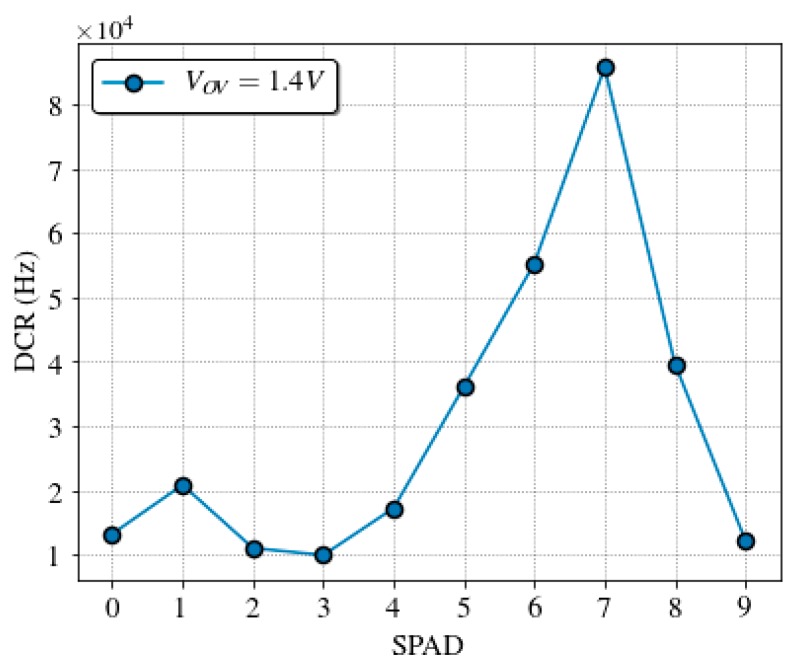
Dark count rate profile (with a maximum relative error of 0.5%) of the used SPAD sensor array at 1.4 V of overvoltage and room temperature.

DCR was measured on 13 sensor chips, to take into account its variability over different SPADs. Figure 9 shows the cumulative plot of the DCR at 1.4 V of overvoltage at room temperature. Almost 70% of the pixels have a dark count rate lower than 8 kHz, which is in line with other works [61,62]. Figure 10 shows a typical dark count profile of the SPAD sensor array at normal operation conditions.

**Figure 11:**
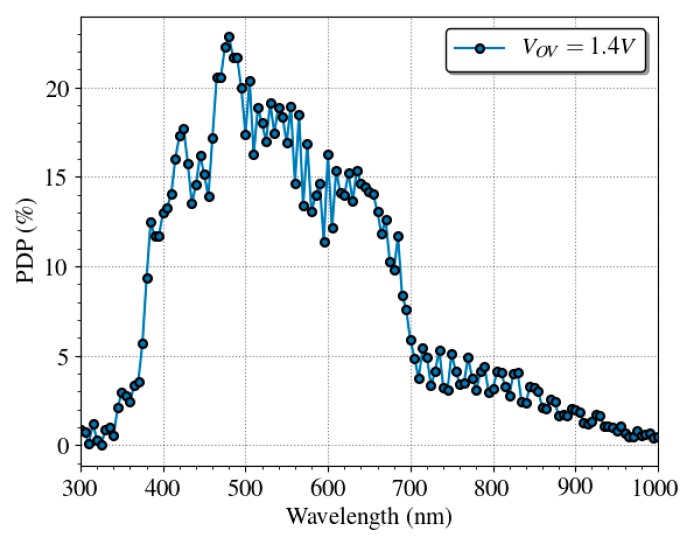
Spectral dependence of the PDP of a single SPAD at 1.4 V of overvoltage with a maximum relative error of 0.5%. The PDP shows interference patterns caused by the passivation layers.

The PDP spectral response of the SPAD has been measured using an electro-optical bench composed by a white-light source, a monochromator filter, and a calibrated reference detector. Figure 11 shows the PDP of a single SPAD in a wavelength range 300–1000 nm for overvoltage of 1.4 V at room temperature. In good agreement, with other works such as [63]. The SPAD presents a good efficiency over the visible range with a maximum of 25% at about 480 nm, and a 15% at the emission wavelength of the QD605, which is 605 nm, at normal operation conditions.

### 3.2. System Performance

Quantum dot samples, QD605 in Phosphate Buffer Saline (PBS), were prepared at concentrations of 1, 1/2, 1/4, 1/8, 1/16, and 1/32 μM. A volume of 0.4 μL of each sample was loaded into the microchannel of the microfluidic cartridge. An effective sample volume of 20 nL (microchannel volume illuminated by the laser spot) was excited to obtain each decay curve in less than a minute. The experiments were carried in normal operation conditions at room temperature in a dark environment, with the SPAD sensor biased at 1.4 V of overvoltage. The dark count rate profile for the SPAD sensor chip used is shown in Figure 10.

**Figure 12:**
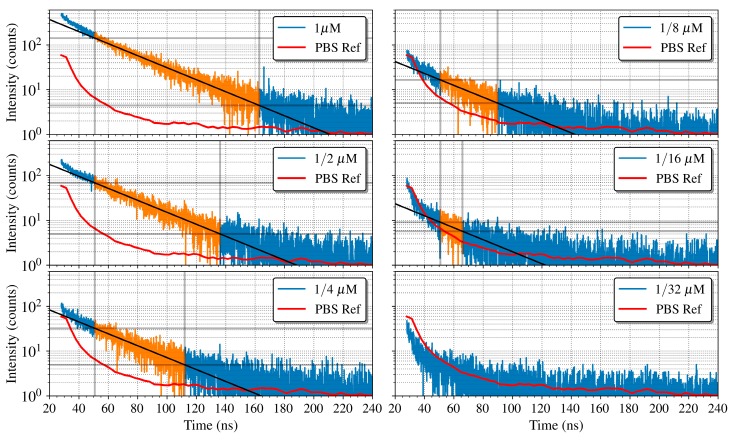
Fluorescence decay curves measured using the SPAD located below the microchannel for different concentrations of QD605 (with a maximum relative error of 0.5%), together with fitting lines used to determine the lifetimes for each concentration. The adjustment lines are calculated using only the points where the predominant effect is the decay of QD605 (highlighted in orange).

A first experiment was done to determine the sensitivity of the PoC-MD. To guarantee the best SNR, the microchannel was fixed on the SPAD with lower noise. The results of the fluorescence decay measurements performed are shown in Figure 12 along with a PBS measurement without quantum dot as a reference. A Savitzky-Golay filter was applied to the PBS reference presented in the figure for clarity in the representation. As expected, the fluorescence intensity decreases with fluorophore concentration. For the 1/32 μM concentration, the decay curve is below the PBS reference, due to the combination of the quantum efficiency, the scattered emission of the QD605 and the absorption of the emitted photons by the neighbour QDs, which reduces the light reaching the active area of the SPAD. The other concentrations follow decay curves above the PBS reference one with three different behaviours. At the beginning, from 20 to 50 ns, the decay curves present higher decay rates because of the laser pulse tail, clearly visible at the beginning of the PBS curve. From the 50 ns mark, the QD605 fluorescence becomes the predominant effect and the decay curves can be approximated to a mono-exponential decay, showing a linear behaviour in a logarithmic representation, as can be seen in the zones highlighted in orange. At last, at the end of the measuring period, the decay rate slows down as it reaches the noise level.

To extract the lifetime, we made a linear fit to the logarithmic representation of the intensity decays on the second region of each curve, setting the cut-off point of the fitted data at three times the noise level. The lifetime is then the inverse of the slope of the resulting line. Lifetimes of 32.4, 32.5, 32.6, 32.7, and 32.4 ns with a 500 ps of error were obtained for the fluorophore concentrations of 1, 1/2, 1/4, 1/8, and 1/16 μM, respectively. These lifetime estimations are in agreement with those in the literature [55,64]. With this set of concentrations of QD605, the detection limit for our system is a concentration of 1/16 μM for both lifetime and intensity. Moreover, to the best of our knowledge, the 1/16 μM is the lowest reported concentration from which a low cost instrument with neither optical lenses nor filters has extracted the fluorescence lifetime [8,15,26].

A second experiment was conducted to study the possibility of using one SPAD regardless of the position of the microchannel over it, as well as checking if it is possible to perform the analysis of two or more samples simultaneously in a microfluidic cartridge with more than one micro channel, labelled with the same fluorophore. To do so, we studied how the detected signal varies with the distance between the SPAD and the microchannel for different concentrations. Taking advantage of the fact that the SPADs have a constant pitch of 50 μm in the array, we placed the microchannel over the first SPAD (number 0) and performed the measurement of the fluorescence intensity and its decay rate for all concentrations with every SPAD on the array.

**Figure 13:**
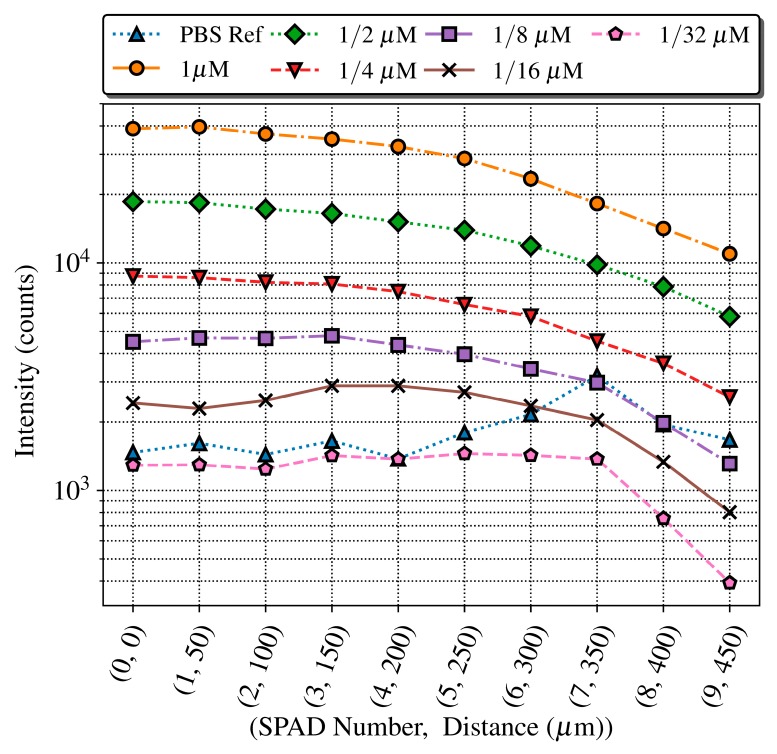
Fluorescence intensity measured across the SPAD array within the time slice 50–75 ns (with a maximum relative error of 0.5%), showing how the level detected decreases with the distance.

Figure 13 shows the fluorescence intensity (total accumulated counts) in the time interval when the predominant contribution is the fluorescence of the QD605 label (from 50 to 75 ns), measured for each SPAD and concentration, as well as the PBS reference. As expected, the intensity of the signal decreases with the distance between the microchannel and the sensor. The PBS reference measurements present a profile similar to the DCR of Figure 10, which sets the detection limit along the SPAD sensor array. The intensity level for the 1/32 μM sample is always below the PBS reference, as expected from the previous experiment. The 1/8 and 1/16 μM concentrations can be detected until the 6th SPAD, meaning that the system operates in optimal conditions with a distance between SPAD and sample of up to 300 μm. Higher concentrations can be detected even with the noisiest SPAD of the array, number seven at 350 μm, which has eight times the noise of the best one.

**Figure 14:**
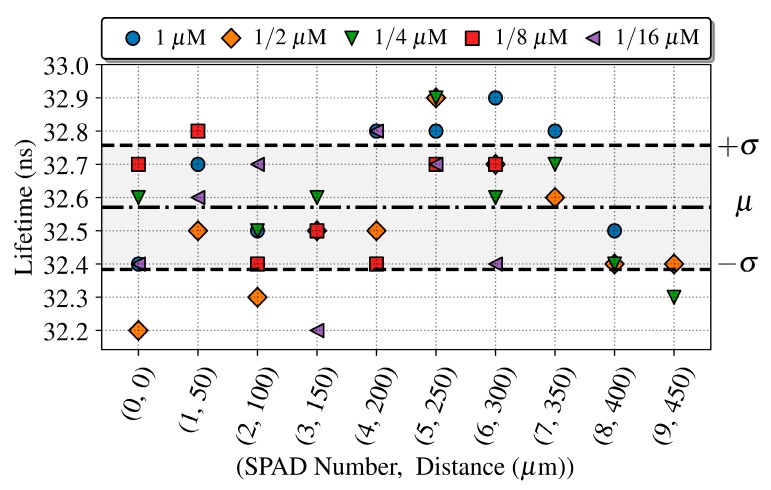
Fluorescent life times of QD605 extracted along the SPAD array for concentrations of 1, 1/2, 1/4, 1/8 and 1/16 μM, with the microchannel placed over the SPAD0. The lifetime estimation error is 500 ps.

Additionally, we calculated the quantum dot lifetime as done for the previous experiment for each concentration and SPAD along the array. Figure 14 shows the extracted lifetimes for the different combinations. As expected, the lifetimes can be extracted only where the detected signal is over the PBS reference signal. The extracted lifetimes are consistent with the previous results along the whole array, with a mean value (μ) of 32.7 ns and a standard deviation (μ ± σ) of 0.2 ns.

In view of the results, we conclude that regardless of where the microchannel falls within the array, it will always be possible to choose among the SPADs one with the sufficient SNR to perform the intensity and lifetime measurements, even in cases in which the microchannel falls out of the array. This gives a good margin of tolerance for the manufacture of the microfluidic cartridge and the SU8 stopper.

Furthermore, the results indicate that it is not possible to analyse two or more samples simultaneously using a single microfluidic cartridge with two or more channels, since they would interfere significantly with the measurements from the others ones. One possibility to explore in order to get around this problem would be to use different fluorophores on each channel, each one decaying according to a different lifetime, and fit a multi-exponential decay on the obtained curves [7]. Such experiment requires fluorophores with long yet different lifetimes, and is out of the scope of the present work. An alternative to the multi-exponential decay analysis might be multiplexing the sample over time. This method consists in reusing the microchannel with different samples, cleaning it after each measurement. This presents two main drawbacks: the absorption of small molecules such as drugs and proteins on the PDMS surface, which has been identified as a major problem for molecular biology [65,66], and the automation of the process, which requires the use of micro-pumps, incrementing the complexity of the setup [27,29]. Further investigation needs to be undertaken to deepen into this issue.

Our PoC-MD builds on the advantages of the cheap single use microfluidic cartridge to offer an elegant and useful solution. The sample can be changed without any alignment procedure, performing the measurement with all the SPADs and analysing the decay curve with best SNR. To prepare the device for industrialization, we need to bring down the size and cost for each device. To do so, we would stop using the expensive commercial development board (such as the Zedboard used in the prototype), moving the control and processing electronics into a custom integrated PCB. We also need an alternative to the PDMS used for the microfluidics, because its production volume is low, of the order of 300–1000 units per month according to Reference [67]. Other materials used to manufacture microfluidics are thermoplastics (like PMMA, COP, PS, PM, COC), but they present autofluorescence emission [68]. The best candidate is a soft ThermoPlastic Elastomer (sTPE), Flexdym, which claims to alleviate PDMS drawbacks in microfluidics (quick prototyping, absorption and substrate bonding) while keeping the advantages (softness, optical properties, biocompatibility and gas permeability) [69]. Flexdym fabrication process can be performed using a hot embossing machine or very simple press equipment, making it compatible with rapid manufacturing technology such as injection molding or roll-to-roll.

## 4. Conclusions

In this paper, we present a fluorescence lifetime spectroscopy PoC for molecular diagnosis that is small, low-cost and easy to assemble, by joining the advantages of a linear array of ultra-sensitive CMOS SPAD-based detectors with a custom PDMS microfluidic cartridge that works with a coarse easy alignment. Moreover, the device avoids the need of any optical filter to remove the excitation light by beginning the measurement process after it has faded below the fluorescence, but that limits the fluorophores to those with long lifetimes, such as the QD605. The SPAD, makes it possible to detect low concentrations of fluorophores thanks to its sensitivity over a wide range of wavelengths.

The results show that the system is able to build the histogram of the fluorescence decay and measure the lifetime from very small sample volumes (20 nL) at practical concentration levels (62.5 nM) in a few seconds. Nevertheless, the detection limit is determined by the noise of the SPAD. Even with the noise limitation, the use of commercial components combined with an ultra-sensitive detector and a removable microfluidic cartridge makes the PoC-MD a low-cost system with great versatility, which holds large potential for applications in an analytical laboratory, clinical diagnosis, at the point-of-care, or in a resource limited environment.

The way towards industrialization of this device has been explored, involving the need to reduce size and cost. PDMS seems to be the more limiting material and a soft thermoplastic elastomer (as Flexdym) appears as the best alternative to PDMS.

## Figures and Tables

**Table 1:** The basic components needed to construct the PoC-MD. It is assumed that the computer, 3D printer, and the facilities to perform soft-lithography and wire bonds are available. The costs for raw materials are divided by the estimated number of devices that can be manufactured with them.

Components	Approx. Cost	Cost Per Device	Notes
SPAD sensor chip (40 dies fabricated in a multi-project wafer)	7000 €	175 €	
Zedboard Zynq-7000 Development Board from Digilent	422 €	422 €	
3D printed spacer (based on the 3D printer cartridge cost (15 €))	0.15 €	0.15 €	
Daughter card (includes all electronic parts and components)	110 €	110 €	
Sensor Packaging			
Substrate based on a printed circuit board	66 €	66 €	
Conductive epoxy CW2400 from Chemtronics Circuit Works	100 €	2 €	
SU-8 100 negative tone near UV photoresist from Microchem Corporation	860 €	8.6 €	1
Laser driver circuit components			
Laser driver circuit DC supply for V+ (includes all electronics parts and components)	96 €	96 €	
Laser diode 405 nm and 150 mW L405P150 from Thorlabs	83 €	83 €	
Microfluidics materials			
Polydimethylsiloxane—PDMS, Dow Corning Sylgard 184 kits	170 €	3.4 €	2
Glass coverslip 12460S from Thermo Fisher Scientific (1000 units)	55 €	0.055 €	
Fluorophore label			
Q10103MP 50 μL 1 μM QdotTM 605 Streptavidin Conjugate from Thermo Fisher Scientific	203 €		
PBS PH7.4 W/O CAMG USA PLASTIC 500 mL	12 €		

1. Around 5 mL are used to encapsulate one SPAD sensor. 2. Around 50 devices can be manufactured with 1.1 kg of PDMS.
